# Commentary: Commentary: Antibodies against multiple post-translationally modified proteins aid in diagnosis of autoimmune hepatitis and associate with complete biochemical response to treatment

**DOI:** 10.3389/fmed.2023.1297191

**Published:** 2023-10-23

**Authors:** Michelle D. van den Beukel, Anna E. C. Stoelinga, Adriaan J. van der Meer, Stef van der Meulen, Lu Zhang, Maarten E. Tushuizen, Bart van Hoek, Leendert A. Trouw

**Affiliations:** ^1^Department of Immunology, Leiden University Medical Center, Leiden, Netherlands; ^2^Department of Gastroenterology and Hepatology, Leiden University Medical Center, Leiden, Netherlands; ^3^Department of Gastroenterology and Hepatology, Erasmus Medical Center, Rotterdam, Netherlands

**Keywords:** pIgG—polyreactive IgG, AIH—autoimmune hepatitis, PTM—post-translational modification, anti-CarP, antibodies against carbamylated proteins, FCS—fetal calf serum, BSA

We read with great interest the commentary of Taubert et al. (1) on our article “Antibodies against multiple post-translationally modified proteins aid in diagnosis of autoimmune hepatitis and associate with complete biochemical response to treatment (2).” In their kind commentary the authors bring up the very important and relevant subject of polyreactive IgG (pIgG) as they have described to occur in autoimmune hepatitis (AIH) (3). The team of Taubert have identified such pIgG using an experimental set up roughly similar to the enzyme-linked immunosorbent assay (ELISA) setup as we have used for the detection of the antibodies against post-translationally modified proteins (anti-PTM). In their commentary they raise the concern that part of the antibodies identified in our assays as anti-PTM antibodies may in fact be pIgG. We can reassure the authors and readers that we are specifically detecting anti-PTM antibodies in our assay. Importantly, this is because of the setup of our ELISA system. Ever since the identification of antibodies binding to carbamylated antigens (anti-CarP) (4) we have used both carbamylated fetal calf serum (Ca-FCS) and unmodified FCS as control antigens for the coating of the ELISA plates. In practice one half of the ELISA plate is coated with Ca-FCS and the other with unmodified, control FCS. The entire plate is blocked with bovine serum albumin (BSA). Each serum sample is tested on both the Ca-FCS and the control FCS. The levels of antibody binding are calculated from absorbance values into arbitrary units per milliliter based on a standard line on the same plate. Next, the level of carbamylation specific antibodies is defined as the level of antibody binding to the Ca-FCS minus the level of antibodies binding to the control FCS. Hence, we report the PTM-specific response. In many of the analyses that we have run for rheumatoid arthritis (RA) and for systemic lupus erythematosus (SLE) (5) the reactivity of the control protein is very low. Indeed, we have observed that in AIH this was somewhat higher, but importantly we have subtracted this from the anti-PTM response, allowing us to conclude on the PTM-specific antibodies and avoiding undesired interference from pIgG. We realize that we may not have stressed this to the greatest extend in our manuscript and thank the authors for bringing up this point and for the opportunity to clarify this.

In our manuscript we have used six different PTMs. To make the best comparisons, we have not used the same control FCS for all the PTMs but have actually generated a separate control FCS for each of the conditions. For example, the control for carbamylation is an aliquot of the same FCS, incubated at the same time point, for the same duration, at the same temperature and dialysis steps as the carbamylated FCS, but only without the addition of the KOCN, the carbamylating chemical. For the modification with Advanced Glycation End-products, we have performed the incubations of the control FCS also for 10 days at 37^o^C, all to ensure that we make the best possible comparisons.

In the original paper we already reported that each of the anti-PTM reactivities has clearly different sensitivities, while all of the assays are based on FCS coating and bovine serum albumin (BSA) blocking, indicating that the assays do not detect pIgG. We have tested if there was any correlation between the signals observed on PTM-FCS vs. control FCS. For the four anti-PTMs with the highest percentage of positive samples we did not find any correlation, again indicating that the anti-PTM antibodies are specifically binding to the PTM. In the absence of PTM specific antibodies there is logically a correlation between the modified FCS and control FCS. The authors raise interesting questions regarding the nature of the antibody response to the PTM proteins. As can be seen in supplementary figure 1 of the manuscript (2), we studied how often the different anti-PTM antibodies can be found together in the same patients, as this may be an indication of either co-induction or cross-reactivity. We clearly observe different patterns with some individuals positive for one anti-PTM and other positive for several others (2), again indicating that the different assays are clearly identifying different antibodies. Additionally, while between some anti-PTM responses we do observe a correlation [as observed before (4, 6)] for other anti-PTM responses we do not detect any correlations. Importantly, some patients can be highly positive for one anti-PTM reactivity and simply negative for the other.

We did find that overall levels of some anti-PTM antibodies (weakly) associate with levels of IgG, but this may simply reflect that a polyclonal B cell stimulation (7) will stimulate the anti-PTM reactive B cells as well as other B cells, but it will only result in positivity in individuals that actually have anti-PTM reactivity. In the context of RA, we have observed that many of the anti-PTM antibodies are isotype switched but are of low-avidity (8, 9) indicating that there has been T-cell help, but lack of avidity maturation. The authors finally raise the point of serum storage time. This is an important issue and difficult to address experimentally. We have previously studied this in detail for our cohort in the context of our previous paper on AIH, focused on other biomarkers (10), where we concluded that the quality of the samples was good as there was no difference in the sensitivity of the markers in the samples that were stored for a long time (i.e., ≥10 years) vs. the samples that were stored more recently (i.e., <10 years), suggesting that the storage was not a major factor in these analyses. Also for the current study on anti-PTM antibodies we have now carefully plotted the levels of all the six anti-PTM reactivities vs. the time of storage of the sample and observed that positivity for the anti-PTM antibodies is not influenced by storage time (data not shown).

Importantly, for the anti-PTM responses in AIH we do observe associations with response to treatment while in the work of Taubert et al. (3) no such association is observed for pIgG, again indicating that the anti-PTM detection does measure different antibodies. For a subset of patients we have analyzed changes in anti-PTM antibody levels over time, and we observed that upon treatment the levels decrease. The data obtained from these two time points does not reveal if the anti-PTM positivity will completely seroconvert.

In conclusion, we agree with the authors of the commentary that unintentional detection of pIgG is an important factor to consider when running ELISA experiments on sera of patients with AIH. However, we are convinced that the careful set up of our experiments excluded the detection of pIgG and specifically measures anti-PTM antibodies.

## Author contributions

MB: Investigation, Writing—original draft, Writing—review and editing, Conceptualization. AS: Conceptualization, Investigation, Writing—original draft, Writing—review and editing. AM: Conceptualization, Writing—original draft, Writing—review and editing. SM: Conceptualization, Investigation, Writing—original draft, Writing—review and editing. LZ: Conceptualization, Investigation, Writing—original draft, Writing—review and editing. MT: Conceptualization, Writing—original draft, Writing—review and editing. BH: Conceptualization, Writing—original draft, Writing—review and editing. LT: Conceptualization, Funding acquisition, Writing—original draft, Writing—review and editing, Supervision.
